# Prenatal Stress and Balance of the Child's Cardiac Autonomic Nervous System at Age 5-6 Years

**DOI:** 10.1371/journal.pone.0030413

**Published:** 2012-01-17

**Authors:** Aimée E. van Dijk, Manon van Eijsden, Karien Stronks, Reinoud J. B. J. Gemke, Tanja G. M. Vrijkotte

**Affiliations:** 1 Department of Public Health, Academic Medical Center - University of Amsterdam, Amsterdam, The Netherlands; 2 Department of Epidemiology, Documentation and Health Promotion, Public Health Service of Amsterdam (GGD), Amsterdam, The Netherlands; 3 Department of Pediatrics, VU University Medical Center, Amsterdam, The Netherlands; University of Sydney, Australia

## Abstract

**Objective:**

Autonomic nervous system (ANS) misbalance is a potential causal factor in the development of cardiovascular disease. The ANS may be programmed during pregnancy due to various maternal factors. Our aim is to study maternal prenatal psychosocial stress as a potential disruptor of cardiac ANS balance in the child.

**Methods:**

Mothers from a prospective birth cohort (ABCD study) filled out a questionnaire at gestational week 16 [IQR 12–20], that included validated instruments for state anxiety, depressive symptoms, pregnancy-related anxiety, parenting daily hassles and job strain. A cumulative stress score was also calculated (based on 80^th^ percentiles). Indicators of cardiac ANS in the offspring at age 5–6 years are: pre-ejection period (PEP), heart rate (HR), respiratory sinus arrhythmia (RSA) and cardiac autonomic balance (CAB), measured with electrocardiography and impedance cardiography in resting supine and sitting positions.

**Results:**

2,624 mother-child pairs, only single births, were available for analysis. The stress scales were not significantly associated with HR, PEP, RSA and CAB (p≥0.17). Accumulation of maternal stress was also not associated with HR, PEP, RSA and CAB (p≥0.07).

**Conclusion:**

Results did not support the hypothesis that prenatal maternal psychosocial stress deregulates cardiac ANS balance in the offspring, at least in rest, and at the age of five-six years.

## Introduction

Growing evidence from multiple disciplines has led to the proposition that the risk of developing coronary heart disease partly depends on environmental factors acting in early life [1], [2]. One of these factors may be psychosocial stress of the mother during pregnancy. Evidence shows that chronic stress conditions significantly influence the maternal hypothalamic-pituitary-adrenal (HPA) axis, leading to hyper secretion of cortisol, which influences the development of the foetal HPA axis [3]–[5].

Less frequently studied is programming of the foetal autonomic nervous system (ANS) by psychosocial stress. Maternal stress may cause a shift towards increased activation of the sympathetic nervous system (SNS) and a decrease in the parasympathetic nervous system (PNS) of the mother, together with increased secretion of catecholamines, all leading to elevated blood pressure and heart rate in the mother [6], [7]. This could have detrimental effects on the development of the foetal nervous system, potentially causing long-term changes in, for example, the sympathetic-parasympathetic nervous system balance, which may lead to cardiovascular diseases at adult age. Increased SNS and decreased PNS activity has been found in foetuses or infants of depressed or anxious pregnant women [8]–[14]. Whether these changes persist throughout childhood is unknown: no human studies including children over 14 months of age have been reported, but animal studies on this subject are convincing [15]–[17].

This study is designed to firstly examine the relation between maternal prenatal psychosocial stress and cardiac ANS balance in the child at age five-six years and to secondly explore potential differences in this relation between boys and girls. We hypothesize that the presence of multiple psychosocial stressors shifts the ANS balance of the child towards increased sympathetic activity, indicated by a shorter PEP, and decreased parasympathetic activity, indicated by decreased respiratory sinus arrhythmia (RSA). The results of this study will provide information on maternal stress as a potential factor working upon the ANS balance in the child, which is important considering the putative role of ANS balance as a causal factor in the development of CVD as well as psychological problems. Moreover, the results may open up new avenues for preventive strategies, given the fact that psychosocial problems during pregnancy are highly prevalent [18].

## Results

With regard to the study population, the characteristics of both the mothers and the children are presented in Table 1.

**Table 1:** Maternal and child characteristics (N = 2,624).

	Mean/Percentage	SD	Interquartile range	
Maternal			Lower	Upper
Age (y)	31.8	4.7	25.8	37.8
Dutch ethnicity (% yes)	75.2			
Pre-pregnancy BMI (kg/m^2^)	22.9	3.8	18.9	26.9
Obesity (BMI ≥30) (% yes)	5.1			
Education after primary school (y)	9.7	3.6	4.7	14.7
Primiparous (% yes)	56.1			
*Hypertension:*				
Pre-existing hypertension (%yes)	3.2			
Pregnancy hypertension (%yes)	8.8			
*Smoking:*				
Non-smoking (% yes)	90.7			
1–5 cigarettes/day (% yes)	6.4			
> = 6 cigarettes/day (% yes)	2.9			
Alcohol (% yes)	26.5			
**Maternal - Stress**				
Depressive symptoms	12	8	2	22
State anxiety	37	10	24	50
Pregnancy-related anxiety (total score)	21	5	16	26
Parenting daily hassles (multiparous, 43.9%)	36	7	26	46
*Job strain:*				
No job (%yes)	25.0			
Low job strain (%yes)	19			
Moderate job strain (%yes)	42			
High job strain (%yes)	14.0			
*Cumulative stress score:*				
No stress (%yes)	52			
1 Stressor (%yes)	31.9			
2 Stressors (%yes)	13			
3–4 Stressors (%yes)	3.1			
**Child – At birth**				
Sex (% boys)	50.3			
Gestational age (weeks)	39.8	1.7	37.9	41.7
Premature (<37 weeks) (% yes)	5.0			
Birthweight (g)	3478	547	2808	4148
*Size at birth:*				
Small for gestational age (% yes)	9.7			
Appropriate for gestational age (% yes)	79.8			
Large for gestational age (% yes)	10.5			
**Child – At age 5 measurement**				
Age (y)	5.7	0.5	5.0	6.5
BMI (kg/m^2^)	15.5	1.5	13.9	17.2

The mean values of the outcome variables, HR, PEP, RSA and CAB, are presented in Table 2, separately for boys and girls. PEP and HR were higher in girls, but their RSA was lower than in boys. CAB did not significantly differ between boys and girls. In both sexes, PEP and HR were higher when sitting up compared with lying down, whereas RSA was lower when sitting up. Only in girls, CAB was significantly different between postures.

**Table 2:** Cardiac autonomic nervous system measures in boys and girls, by posture (N = 2,624).

	Boys						Girls				
	Lying down			Sitting up			Lying down		Sitting up		
	Mean	SD		Mean	SD		Mean	SD	Mean	SD	
Heart rate (bpm)	83.7	9.3	*	89.3	9.9	* †	86.7	9.8	92.4	10.2	†
Pre-ejection period (msec)	76.6	11.6	*	78.5	12.2	* †	77.6	10.1	81.1	11.6	†
Respiratory sinus arrythmia (msec)	127.8	59.5	*	114.9	53.9	* †	121.6	56.3	108.2	51.0	†
Cardiac Autonomic Balance	−0.01	1.46		−0.06	1.39		−0.03	1.36	0.03	1.33	†

*p<0.05 for one sample T-test on sex difference.

† p<0.05 for paired samples T-test on posture difference.

Depressive symptoms, state anxiety, pregnancy-related anxiety and parenting daily hassles were not significantly associated with HR, PEP, RSA and CAB, both in the minimally adjusted (model 1) and the fully adjusted models (model 2) (p≥0.08) (Table 3). Of the individual stress scales, only job strain was associated with one outcome variable: PEP was 1.5 msec lower in the high job strain category (p = 0.03).

**Table 3:** Associations between stress instruments and cardiac autonomic nervous system measures (N = 2,624).

	Model 1	Model 2	Model 1	Model 2	Model 1	Model 2	Model 1	Model 2
	HR		PEP		RSA		CAB	
Depressive symptoms (β)	0.0	0.0	0.0	0.0	0.1	0.1	0.00	0.00
State anxiety (β)	0.0	0.0	0.0	0.0	0.1	0.0	0.00	0.00
Pregnancy-related anxiety (β)	0.1	0.0	0.0	−0.1	−0.3	−0.2	−0.01	0.00
Parenting daily hassles (β) <$>\scale 90% \raster="rg1"<$>	0.0	0.0	0.0	0.0	−0.1	−0.1	0.00	0.00
*Job strain*								
Low job strain (ref)								
No job (β)	0.4	0.3	−0.6	−0.8	−2.0	−4.2	−0.09	−0.15
Moderate job strain (β)	−0.5	−0.3	−0.5	−0.5	−1.3	−1.5	−0.07	−0.08
High job strain (β)	−1.0	−0.8	−1.5 **	−1.5 **	6.4	5.3	−0.02	−0.04

	Model 1	Model 2	Model 1	Model 2	Model 1	Model 2	Model 1	Model 2
	HR		PEP		RSA		CAB	
*Cumulative stress score*								
No stress (ref)								
1 Stressor (β)	0.7	0.6	−1.0 **	−1.1 **	−2.2	−2.7	−0.13 **	−0.14 **
2 Stressors (β)	−0.2	−0.7	−0.8	−0.7	3.7	2.5	0.00	−0.01
3–4 Stressors (β)	−0.3	−0.7	−0.8	−0.9	8.3	5.6	0.09	0.03

Multivariate associations were explored using mixed models to include cardiac autonomic nervous system measures from both the supine and sitting up position (multilevel regression analyses).

* To model 1, sex and age of the child at measurement were added as covariates.

† To model 2, additionally added covariates are: maternal age, ethnicity, pre-pregnancy BMI, educational level, primiparity, hypertension, smoking, alcohol consumption, gestational age and birth weight.

<$>\vskip -2\scale 60% \raster="rg1"<$> Only analyzed in multiparous women (N = 1,147).

**p<0.05.

Overall, the cumulative stress score was not associated with HR, PEP, RSA and CAB, both in the minimally adjusted (p = 0.18; 0.12; 0.15 and 0.08 respectively) and the fully adjusted models (p = 0.07; 0.12; 0.26 and 0.07 respectively). However, the 1 stressor-category was associated with 1.1 msec lower PEP (p = 0.02) and 0.14 lower CAB (p = 0.01) in the fully adjusted models.

Sex of the foetus was not an effect-modifier in the association between any of the stress measures and the ANS measures (p>0.08 for all outcomes and stress measures, data not shown).

Additionally, an interactive effect of maternal stress and posture in which ANS activity was tested. In three cases, the interaction term reached statistical significance (data not shown): In the depressive symptoms – HR model (p<0.01), the pregnancy-related anxiety – HR model (p = 0.03) and the job strain – HR model (p = 0.01). Subsequently, the two-level mixed model was stratified into separate linear regressions for HR whilst lying down and HR whilst sitting up. The associations between these stress measures and HR however remained statistically insignificant (p>0.26 for all stress measures, data not shown).

## Discussion

From this study it appears that prenatal maternal psychosocial stress is not associated with multiple measures of the child's ANS in rest at the age of five-six years.

In contrast to the current results, previous studies did observe such associations. Those study populations however, consisted of younger offspring exposed to maternal stress during pregnancy. Both Monk et al. and DiPietro et al. observed associations of maternal mood, stress and anxiety with intrauterine foetal heart rate in relatively small study samples [8]–[10]. Similar results have been reported in neonates: A higher number of maternal life stressors was associated with lower heart rate variability [11] and maternal depression was also associated with lower vagal tone in newborns [12], [13]. Both are indicators of reduced cardiac parasympathetic activity. Dierckx et al. observed reduced vagal tone and increased heart rate, also indicators of reduced parasympathetic nervous system activity, in 14-month old infants of mothers with a lifetime psychiatric diagnosis in a relatively large (N = 528) study sample. Their data do not however support an effect of just prenatal psychiatric status, besides lifetime psychiatric diagnosis, on infant ANS [14].

The method of assessment of maternal stress varies greatly between similar studies. We chose to use multiple validated psychosocial stress constructs as well as a cumulated score in order to identify women subjected to high levels of stress occurring in normal pregnant women's day-to-day lives. Although in the original ABCD cohort the occurrence of psychosocial stressors was higher than it is in the current group participating 5–7 years later, 3% of the current study population still reported 3–4 stressors. If high levels of psychosocial stress would have influenced the ANS at rest in the offspring, we would at least have expected a trend in that direction.

The timing of stress assessment may also be key to finding potential foetal programming effects. In the current study, the assessment of stress took place near the end of the first trimester, which is often considered the trimester with the highest foetal vulnerability [19]. Some studies have illustrated that, as pregnancy advances, women become decreasingly sensitive to the effects of stress [20], while others observed associations between depressive symptoms in the second and third trimester with reduced neonatal vagal tone [12], [13]. PD Wadhwa has repeatedly underscored the need for more attention to be paid to the timing of stress in future studies, because there are possibly critical periods of vulnerability that remain unclear to date (e.g. [21], [22]).

The cardiac ANS measurements took place outside a laboratory environment using a validated ambulatory device [23], allowing us to include a large number of subjects. The observed values, including the boy vs. girl and lying down vs. sitting up differences were in line with what would be expected from the literature [24], [25]. One exception was the higher average PEP-value in upright position as compared to the supine position. This is likely due to the large afterload effects induced by postural changes, which elongate PEP by prolonging the time needed to open the aortic valve [26]. As we did our comparisons within postures, the PEP would still be a valid indicator for sympathetic cardiac drive.

Prenatal maternal stress has been associated with small for gestational age births or intrauterine growth retardation [27]. In agreement with previous studies [25], [28], [29], decreasing birthweight was in our data associated with higher heart rate. Apparently prenatal factors other than maternal stress, related to intrauterine growth retardation, have the potential to program the offspring's ANS.

It could be argued that some factors may have influenced our results. First, the current population's diverse ethnicities may be a factor in the association of both maternal psychosocial stress and the cardiac ANS measures, because ethnic differences in ANS have been reported [30], [31]. However, results from the Dutch women only did not change the results (data not shown). Second, the child's BMI at age five-six was associated with both maternal psychosocial stress and all four of the cardiac ANS measures. We did not adjust for this variable in the analyses because of its possible mediating effect. We do not expect this to cause confounding, since the relation of stress and ANS was not observed. Third, maternal pre-existing hypertension may also be a mediating mechanism between prenatal stress and the offspring's ANS, because literature suggests that stress is associated with higher blood pressure [32]. Our fully adjusted model did, however, incorporate maternal hypertension just as a confounder, because we were interested in mediation via early life factors. The results showed that even in the unadjusted model, maternal stress and the child's ANS were not associated.

An explanation for our null-finding could be that we only measured the children in rest. It is possible that prenatal stress does not have an effect on baseline values, but only an effect on the set points for stress reactivity, causing hyper reactivity. For example, increased cardiovascular reactivity to stress was observed in babies of smokers and babies born preterm [33] and increased cardiac sympathetic activation was observed in babies who were small at birth [25], [29]. Future studies regarding prenatal stress should consider reactivity, subjecting the offspring to a stress test when assessing ANS functioning.

Also, as mentioned above, the method of assessment of psychosocial stress, or even the definition of psychosocial stress itself, is not agreed upon in literature. Some studies observed impact on offspring when studying depressive symptoms [12], [13] whereas others studied lifetime psychiatric diagnosis [14]. Our focus on phasic psychosocial stress may not have sufficient impact on the offspring, offering an alternate explanation for our null-finding.

Another possible explanation for the absence of an association in the current study can be sought in the child's age. The children in previous studies with significant findings were younger [8]–[14]. Perhaps the differences in ANS between offspring exposed and not-exposed to prenatal stress have attenuated. Or, in contrast, maybe the differences are getting stronger again with ageing of the child, as daily life stressors could have stronger effects in the offspring of stressed mothers by higher and more frequent responses of the ANS.

In conclusion, prenatal maternal psychosocial stress does not appear to be associated with multiple measures of the child's cardiac autonomic nervous system in rest at the age of five-six years. Future studies may focus on ANS reactivity during validated stress-protocols, and on the maturation of the ANS under the influence of daily life stressors experienced by the child in longitudinal follow-up studies.

## Materials and Methods

The present study is part of the Amsterdam Born Children and their Development (ABCD) study, a prospective, longitudinal birth cohort [34]. Approval was obtained from the Academic Medial Center Medical Ethical Committee, the VU University Medical Center Medical Ethical Committee and the Registration Committee of Amsterdam. All participating mothers gave written informed consent for themselves and their children.

### Study population

During 2003–2004, 12,373 Amsterdam women who first attended antenatal care in Amsterdam were approached to participate in the ABCD study. Of these women, 8,266 (67%) returned the pregnancy questionnaire, which covered sociodemographic characteristics, obstetric history, lifestyles and emotional problems (including multiple psychosocial stress instruments) (phase 1). In the following years, the ABCD study covered a questionnaire around three months after birth and the follow-up of growth from the Youth Health Care Centers (phase 2). The ABCD study has been described in detail elsewhere [34].

**Figure 1:**
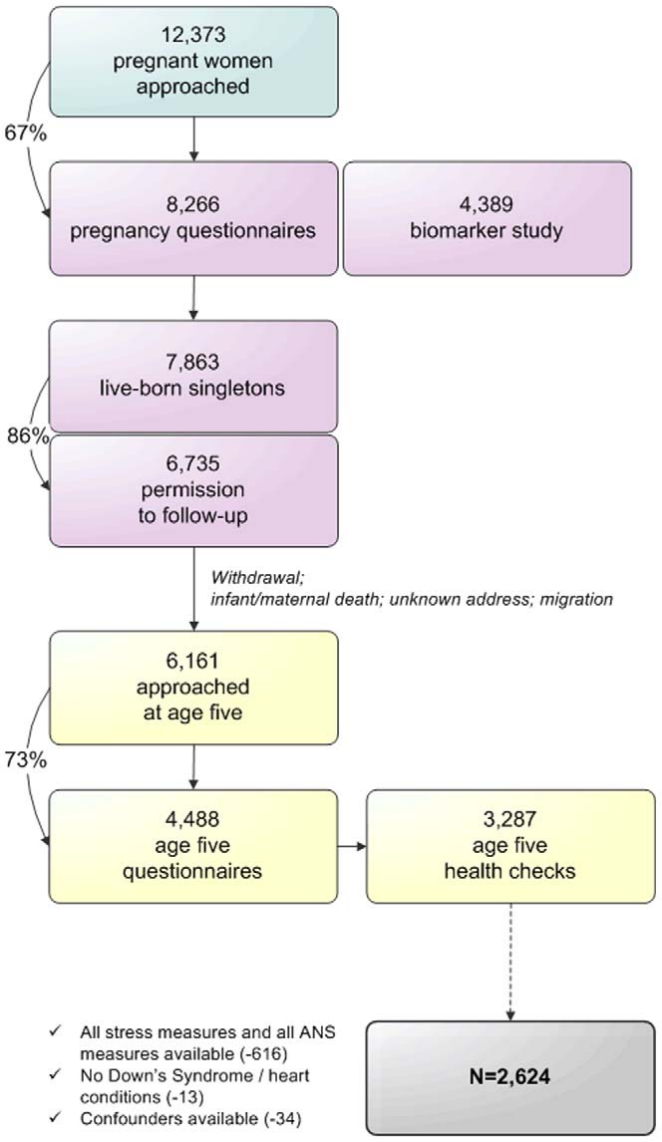
Procedure of the ABCD Study cohort and inclusion in the current analyses.

In 2008, when the first children had reached the age of five years, the third phase of the study started. The addresses of 6,161 mothers were retrieved from the Youth Health Care registry. The mothers received a questionnaire, including an informed consent form for the age five health check, which was returned by 4,488 (73%) of the participants. The health check itself consisted of various health measurements in 3,287 children aged five to seven years (2008–2010; mean age 5.7 years ± 0.5 SD). Multiple births were excluded from the cohort preceding the third phase. The selection of the population included in the current study's analyses (N = 2,624) is visualized in Figure 1.

### Independent variables: maternal prenatal psychosocial stress

In the selected population, the pregnancy questionnaire including the psychosocial stress constructs was filled out at gestational week 16 [IQR 12–20].

#### State anxiety

Anxiety was assessed using the Dutch version [35] of the State-Trait Anxiety Inventory (STAI) [36]. The 20 items regarding state anxiety (transient or temporarily experienced anxiety) were included in our questionnaire, with each item scored on a 4-point scale (0 = rarely or none of the time, 1 = some or a little of the time, 2 = occasionally or a moderate amount of the time and 3 = most or all of the time).

#### Depressive symptoms

Depressive symptoms were assessed using the validated Dutch version of the 20-item Center for Epidemiological Studies Depression Scale (CES-D) [37], [38]. This scale evaluates the frequency of depressive symptoms experienced over the preceding week. Each item was scored on a four-point scale (0 = rarely or none of the time, 1 = some or a little of the time, 2 = occasionally or a moderate amount of the time and 3 = most or all of the time).

#### Pregnancy-related anxiety

Pregnancy anxiety was assessed using an abbreviated 10-item version [39] of the Pregnancy Related Anxieties Questionnaire (PRAQ) [40]. Each item was scored on a four-point scale (0 = definitely not true, 1 = not true, 2 = true and 3 = very true). Three aspects that can be distinguished are ‘fear of giving birth’, ‘fear of bearing a physically or mentally handicapped child’ and ‘concern about one's appearance’, but in the current study only the overall score was utilized.

#### Parenting stress

To assess parenting stress a Dutch adaptation [41] of the 20-item Parenting Daily Hassles (PDH) scale was used [42]. The parents rated the occurrence of typical everyday events in parenting and parent-child interactions on a four-point scale (0 = never or rarely, 1 = sometimes, 2 = a lot and 3 = constantly). The ABCD pregnancy questionnaire did not include the accompanying PDH hassle-scale. Women with no previous children scored zero on this scale by default.

#### Job strain

To assess job strain (or work stress), a Dutch version of the Job Content Questionnaire was used [43], [44]. It consists of 2 subscales: job demands and job control. The job demands subscale consists of 25 four-point scale items focusing on work pace, mental workload and physical workload. The job control subscale consists of 11 items. The total score of the job demands scale was dichotomized using the 80th percentile as cut off and the job control scale using the 20th percentile as cut-off to create four (2×2) categories of job strain. Jobs that are high in demands and low in control are considered most stressful (high job strain).

#### Cumulative stress score

A total, cumulative stress score was calculated by ascribing points to the number of times a mother ends up above the 80th percentile of three of the above mentioned stress scales (depressive symptoms, pregnancy-related anxiety and parenting stress). A fourth point is added if the mother also scored high on the job strain scale. This resulted in a sum score between 0 and 4, which was divided into three categories: No stress (0 stressors), 1 stressor, 2 stressors, and 3–4 stressors. State anxiety was not included in the total score because of its high correlation with depressive symptoms (correlation coefficient 0.9).

### Dependent variable: cardiac autonomic nervous system activity of the child

Cardiac autonomic nervous system activity was assessed at the age five health check, using an ambulatory device, the VU University Ambulatory Monitoring System (VU-AMS; Amsterdam, the Netherlands). Reliability and validity aspects and recording methodology of the VU-AMS have been described previously [23]. The system records three lead electro cardiograms (ECG) and four lead impedance cardiograms (ICG) (Ultratrace Diagnostic ECG with wet gel; ConMed Corporation, Utica, New York, United States of America).

The procedure of this measurement has been extensively described previously [45]. In short, to start with, the child was lying down in a supine position for one minute of stabilization, after which registration was started for about six minutes. Next, the child was seated at a table for one minute of stabilization followed by about six minutes of registration.

All R-peaks in the ECG, scored by the software, were checked and R-peak markers were moved, inserted or deleted. The software also automatically marked inspirations and expirations in the respiratory signals, which were also checked, but no edits were necessary. From the resulting data, RSA was automatically obtained as a derivate of parasympathetic nervous system activity, a time domain index of heart rate variability in the respiratory frequency range [46]. RSA is the peak valley estimation (pvRSA) which was obtained automatically by subtracting the shortest inter beat interval during heart rate acceleration in the inspirational phase from the longest inter beat interval during deceleration in the expirational phase.

As a derivate of sympathetic nervous system activity, PEP was used. PEP is the time interval between the onset of ventricular depolarization (the Q wave onset in the ECG) and the opening of the aortic valves (B-point in ICG) and is considered to be an adequate surrogate measure for cardiac sympathetic nervous system activity [23]. It was scored manually in large-scale ensemble averages of the impedance cardiograms [47]. These two measures can be combined to form the cardiac autonomic balance (CAB = zRSA−(−zPEP) [48]). Low values reflect parallel high sympathetic and low vagal (parasympathetic) cardiac control, which is therefore unfavourable. In sum, outcome measures HR, PEP, RSA and CAB were assessed.

### Potential confounders

Maternal age, ethnicity, pre-pregnancy BMI (kg/m^2^), educational level, smoking and alcohol consumption were available from the pregnancy questionnaire, hence all self-reported. Ethnicity was defined by maternal country of birth (in line with the definition by Statistics Netherlands (CBS). Education after primary school was defined in years and considered a proxy for socioeconomic status. Smoking during pregnancy was categorized into non-smoking, 1–5 cigarettes/day and > = 6 cigarettes/day. Alcohol consumption during pregnancy was dichotomized (yes/no).

Hypertension (no/pre-existent/pregnancy-induced) was available by combining data from the questionnaire and Dutch Perinatal Registration (PRN; www.perinatreg.nl). The categories were classified in accordance with the guidelines of the International Society for the Study of Hypertension in Pregnancy (www.isshp.com).

Parity (primiparous: yes/no), gestational age, birth weight and sex were available from the PRN and Youth Health Care Registration. Gestational age was additionally dichotomized into at term and preterm (gestational age <37 weeks). To define ‘birth size’, the bottom and top 10% of birth weight, standardized for gender, gestational age and parity using reference values from PRN, were labelled small-for-gestational-age and large-for-gestational-age respectively. The middle 80% was labelled appropriate-for-gestational-age.

The BMI of the child was measured at the age five health check. Height was determined to the nearest millimetre using a Leicester portable height measure (Seca, Hamburg, Germany) and weight to the nearest 100 gram using a Marsden MS-4102 weighing scale (Oxfordshire, United Kingdom).

### Statistics

Associations were explored using mixed models to include cardiac autonomic nervous system measures from both the supine and sitting up position (multilevel regression analyses) (SPSS 18.0, SPSS Inc., Chicago, USA). Regression analyses were standardized for sex and age of the child by default. All potential confounders were determined a priori and added simultaneously. Effect-modification was tested by adding an interaction term. Statistical significance (two-sided) was determined at α = 0.05.
